# Endovascular flow-diversion of visceral and renal artery aneurysms using dual-layer braided nitinol carotid stents

**DOI:** 10.1186/s42155-020-00125-2

**Published:** 2020-06-28

**Authors:** Penelope van Veenendaal, Julian Maingard, Hong Kuan Kok, Dinesh Ranatunga, Tim Buckenham, Ronil V. Chandra, Michael J. Lee, Duncan Mark Brooks, Hamed Asadi

**Affiliations:** 1 Interventional Radiology Unit - Monash Imaging, Monash Health, Melbourne, Australia; 2 Interventional Neuroradiology Unit, Monash Health, Melbourne, Australia; 3 School of Medicine – Faculty of Health, Deakin University, Waurn Ponds, Australia; 4 Monash Hospital, Clayton, Victoria Australia; 5 Interventional Radiology Service – Department of Radiology, Northern Hospital, Melbourne, Australia; 6 Interventional Radiology Service - Department of Radiology, Austin Hospital, Melbourne, Australia; 7 Department of Imaging, Monash University, Melbourne, Australia; 8 Interventional Radiology Service – Department of Radiology, Beaumont Hospital, Dublin, Ireland; 9 Royal College of Surgeons in Ireland, Dublin, Ireland; 10 Interventional Neuroradiology Service – Radiology Department, Austin Hospital, Melbourne, Australia

**Keywords:** Dual layer, Stent, CASPER, Aneurysm, Visceral, Renal, Splenic

## Abstract

**Background:**

Visceral and renal artery aneurysms (VRAAs) are uncommon but are associated with a high mortality rate in the event of rupture. Endovascular treatment is now first line in many centres, but preservation of arterial flow may be difficult in unfavourable anatomy including wide necked aneurysms, parent artery tortuosity and proximity to arterial bifurcations. Endovascular stenting, and in particular flow-diversion, is used in neurovascular intervention to treat intracranial aneurysms but is less often utilised in the treatment of VRAAs. The CASPER stent is a low profile dual-layer braided nitinol stent designed for carotid stenting with embolic protection and flow-diversion properties. We report the novel use of the CASPER stent for the treatment of VRAAs. We present a case series describing the treatment of six patients with VRAAs using the CASPER stent.

**Results:**

Six patients with unruptured VRAAs were treated electively. There were three splenic artery aneurysms and three renalartery aneurysms. Aneurysms were treated with the CASPER stent, with or without loose aneurysm coil packing or liquid embolic depending on size and morphology. All stents were successfully deployed with no immediate or periprocedural complications. Four aneurysms completely occluded after serial imaging follow up with one case requiring repeat CASPER stenting for complete occlusion. In one patient a single aneurysm remained patent at last follow up, A single case was complicated by delated splenic infarction and surgical splenectomy.

**Conclusion:**

Preliminary experience with the CASPER stent suggests it is technically feasible and effective for use in the treatment of VRAAs.

## Introduction

Visceral and renal arterial aneurysms (VRAAs) are rare, with their incidence estimated at 0.1–2% (Berceli 2005; Pasha et al. 2007; Belli et al. 2012). Most VRAAs are detected incidentally. Elective treatment of patients at high risk of aneurysm rupture is preferred as presentation with rupture is associated with a high mortality rate (Shanley et al. 1996; Carr et al. 1996).

Most true aneurysms occur due to degenerative atherosclerosis. Pseudoaneurysms are most commonly iatrogenic, with other causes including trauma, pancreatitis, liver transplant, mycotic aneurysms, vasculopathy and tumours (Pitton et al. 2015).

Indications for treatment of true VRAAs are symptomatic aneurysms, size > 2.0 cm in diameter, increasing size on surveillance imaging, planned organ transplantation or donation and in women of childbearing age (Ha et al. 2009). All pseudoaneurysms are generally treated regardless of size due to a very high risk of rupture and subsequent mortality (Tulsyan et al. 2007). With the decreased morbidity associated with endovascular treatment, especially as it becomes more routine, the risk: benefit ratio has changed treatment thresholds.

Endovascular treatment involves reconstructive techniques with parent artery preservation with the use of stent-graft exclusion or stent assisted coiling, or deconstructive techniques with sacrifice of the parent artery with coils deployed proximal and distal to the aneurysm neck. The most common endovascular techniques utilize platinum coils, stent-graft placement, segmental vascular exclusion, N-butyl cyanoacrylate (NBCA), Onyx, thrombin or a combination of techniques (Loffroy et al. 2015; Spiliopoulos et al. 2012). Flow-diversion is a novel technique for VRAAs in the peripheral and visceral circulation, which has been adapted from neurovascular treatment of intracranial aneurysms.

The CASPER carotid stent (MicroVention, CA, USA) is a dual-layer stent with an internal micromesh designed for embolic protection and a braided nitinol design to minimize kinking and conform to arterial anatomy with resulting flow diverting properties. It is currently licenced for treatment of internal carotid artery stenosis (Yamada et al. 2017; Wissgott et al. 2015; Diaz et al. 2018). The braided micromesh stent design renders some flow-diversion properties. These features, combined with a range of stent sizes suited to visceral arteries, makes the CASPER stent an attractive option for the treatment of VRAAs in tortuous or unfavourable anatomy and allows for side-branch preservation where supply to the end-organ is critical.

We present the initial experience, feasibility and short-term results of using the CASPER stent to treat six cases of VRAAs, including three splenic artery aneurysms and three renal artery aneurysms.

## Materials and methods

Six cases with unfavourable anatomy which would be difficult to manage with more conventional endovascular techniques were selected in a multidisciplinary setting after review of multimodal imaging including computed tomography angiography (CTA). Informed consent and aseptic technique were employed for all procedures. All patients were commenced on dual antiplatelet therapy including 100 mg Aspirin and 75 mg Clopidogrel 1 week prior to the described procedure. Detailed technical discussion follows in the individual case descriptions. Initial follow-up imaging with either CTA or ultrasound was performed at 4–6 weeks with serial follow up imaging as appropriate.

## Results

Table 1 outlies the clinical and imaging features, technical aspects, complications and follow up in all six cases.

**Table 1:** Baseline imaging features and treatment approaches

Patient	Indication	Size and morphology	Aneurysm close to major branch to be preserved	Tortuosity	Wide neck	Techniques	Complications	Follow up
1	Splenic artery aneurysm > 2 cm	39 mm, bilobed	Yes	Severe	Yes	CASPER 9x30mm and coil embolization of aneurysm sac	No	Complete occlusion at 19 months on Doppler ultrasound
2	Two splenic artery aneurysms > 2 cm in post liver transplant patient	Distal 25 mm, saccular, proximal 28 mm sidewall aneurysm	No	Moderate	Yes	CASPER 7x30mm (distal) and coil embolization (proximal) – some concerns with initial stent deployment	No	Complete occlusion on CTA at 13 months post the initial treatment following delayed procedure with deployment of an additional CASPER
3	Splenic artery aneurysm > 2 cm	28 mm, bilobed	No	Severe	Yes	CASPER 8x40mm and coiling of medial branch vessel	Infected splenic infarct resulting in open splenectomy	
4	Renal artery aneurysm > 1.5 cm	31 mm, right renal artery aneurysm	Yes	Mild	Yes	CASPER 9x30mm	No	Reduced size, partially thrombosed on 2 month CTA. Complete aneurysm thrombosis at 12 months ultrasound
5	Renal artery aneurysm > 1.5 cm	24 mm, left renal artery bifurcation aneurysm	Yes	Mild	Yes	CASPER 7x18mm	No	Partial sac thrombosis at 12 month CTA Maximum diameter of flowing component reduced from 24 to 15 mm.
6	Renal artery aneurysm > 1.5 cm	49 mm, right renal artery bifurcation aneurysm	Yes	Mild	Yes	CASPER 7 × 25 mm and Onyx HD-500	No	Complete thrombosis of aneurysm sac on CTA at 3 months

### Patient 1

**Fig. 1:**
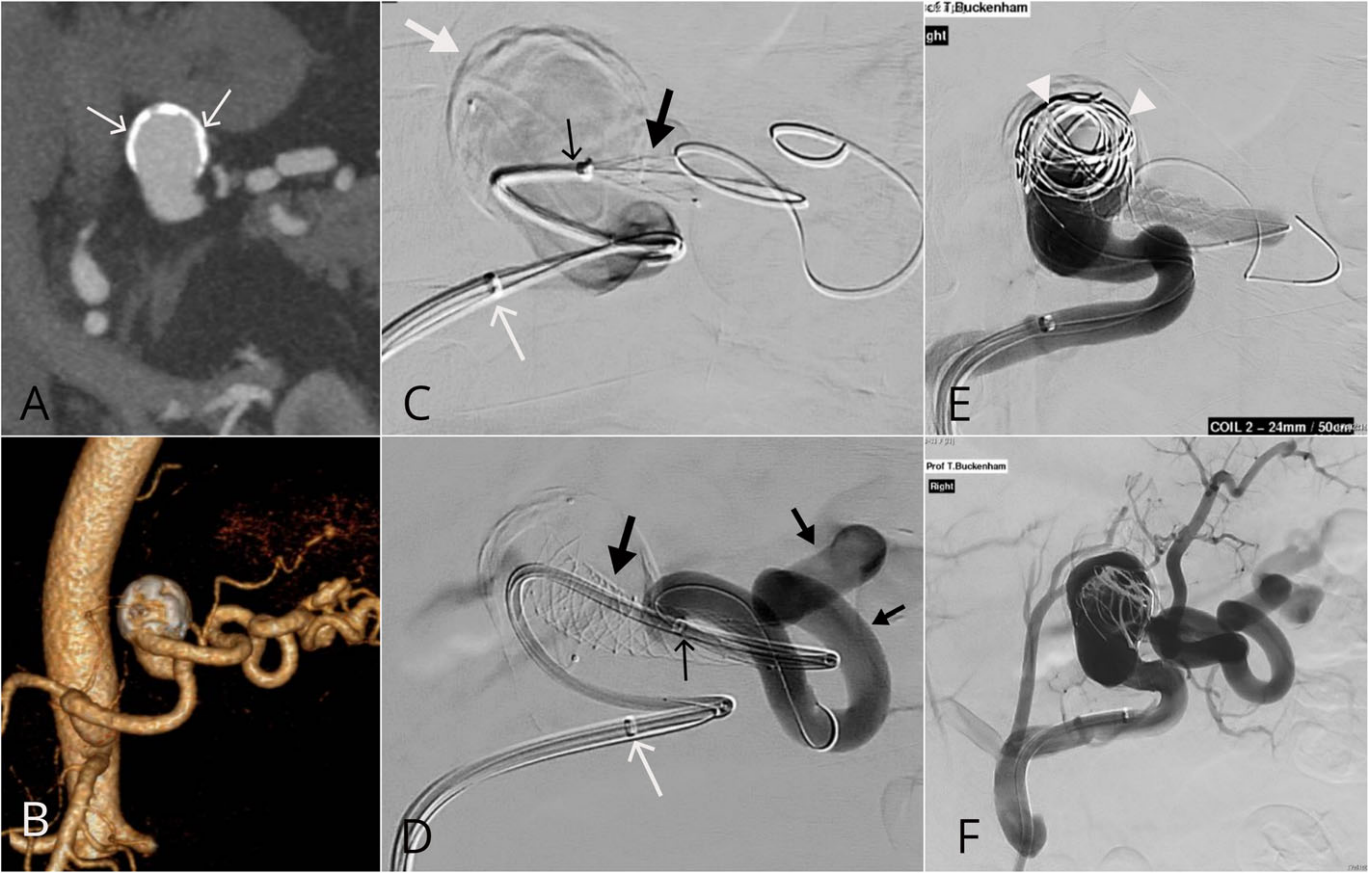
Large wide necked splenic artery aneurysm. **a** and **b** An incidentally detected 39 mm partially calcified wide (white arrows) necked (14 mm) splenic aneurysm was seen to arise from the midportion of a tortuous splenic artery. **c** after difficulty obtained stable access using a 6Fr NeuronMAX 088 guide sheath (thin white arrow) the aneurysm was accessed with a Headway Duo microcatheter and the CASPER stent (thick black arrow) deployed over an 0.014 in. microwire. The marker of the CASPER deployment system (thin black arrow) can be seen during unsheathing of the stent. **d** The 6Fr NeuronMAX 088 guide sheath remained stable in position (thin white arrow) after CASPER stent deployment (thick black arrow). A 5Fr Sofia intermediate catheter was used to navigate through the stent. An angiogram performed via the 5Fr Sofia demonstrates patency of the distal splenic artery (small black arrows). **e** 24 mm coils were subsequently loosely packed into the aneurysm to promote thrombosis (white arrow heads). **f** The final angiogram demonstrates patency of the stent and distal splenic artery and its branches with stasis seen within the aneurysm sac

**Fig. 2:**
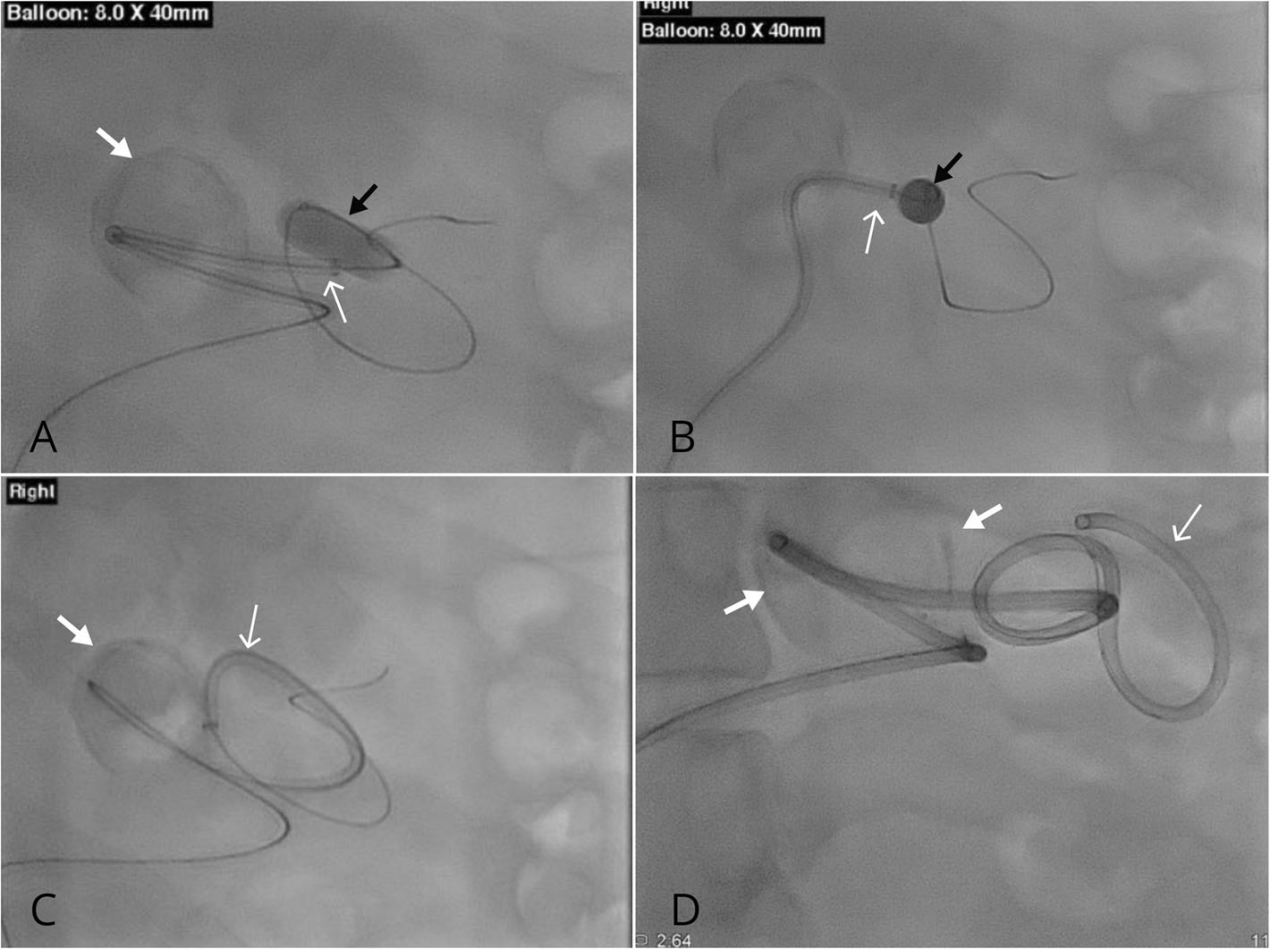
Balloon anchoring technique. **a** – **d** Sequential images demonstrate successful utilisation of the balloon anchoring technique. Initial access was obtained using a 5Fr diagnostic catheter and 0.035 hydrophilic wire. An 8.0 × 40 mm Armada balloon (black arrow) was subsequently inflated and used as an anchor to advance the 6Fr NeuronMAX 088 guide sheath (thin white arrows). Note the partially calcified splenic artery aneurysm (thick white arrows)

A 49-year-old male presented for investigation of right flank pain. There was an incidental finding of a bilobed aneurysm arising from the mid splenic artery measuring 2.6 × 3.6 × 3.9 cm deemed suitable for endovascular treatment. There was significant splenic artery tortuosity and the aneurysm arose at an unfavourable angle with a wide neck morphology (Fig. 1a and b). Under conscious sedation, 8-french right common femoral artery access was obtained. An 8.0 × 40 mm angioplasty balloon (Armada, Abbott Vascular) was used as an anchor to advance a 6-French Neuron MAX 088 guide sheath (Penumbra Inc., CA, USA) in the outflow artery. One 9x30mm and two 10 × 30 mm CASPER stents were placed from the outflow through the aneurysm sac into the inflow artery (Fig. 1c and d). Subsequently, two MicroPlex18 24 mm Cosmos Complex coils and One Hydrofill 24 mm Helical coil were placed into the sac through a jailed Headway Duo microcatheter (MicroVention, Tulsin, CA) (Fig. 1e). A post-procedural angiogram demonstrated satisfactory stent position and patency of the distal artery with stasis within the aneurysm sac (Fig. 1f). There were no immediate complications. Follow-up ultrasound performed at 1 month, 7 months and 19 months post-procedure demonstrated no flow into the aneurysm sac and normal waveforms in the splenic artery. The balloon anchoring technique used during this case is outlined in Fig. 2.

### Patient 2

A 50-year-old male with a history of a liver transplant for hepatitis C cirrhosis presented with two incidentally detected saccular splenic artery aneurysms. A proximal splenic artery aneurysm measured 28 × 20 × 23 mm and a distal aneurysm measured 25 × 22 × 18 mm. The distal wide necked aneurysm arose from a tortuous segment of the parent splenic artery potentially complicating the introduction of a standard stent graft. The wide neck was felt to increase the risk of coil protrusion from the aneurysm sac into the parent artery and thus increased the risk of thromboembolic complications and treatment failure. Under conscious sedation, an 8-French sheath (Terumo, Tokyo, Japan) was inserted to the right common femoral artery and the parent splenic artery accessed using a 6-French Neuron MAX 088 guide sheath (Penumbra Inc., CA, USA). Subsequently, the distal splenic artery aneurysm was crossed using a 6-French FARGOMAX (Balt Extrusion, Montmorency, France) and a 7x30mm CASPER stent was deployed. There were some concerns with positioning of the stent however significant parent vessel tortuosity made re-sheathing of the partially deployed stent difficult and positioning was subsequently deemed acceptable given the technical difficulties. The proximal splenic artery aneurysm was coil embolised with detachable coils (Stryker, MI, USA) using a balloon assisted coiling technique with a Headway Duo microcatheter (MicroVention, Tulstin, CA) and 10 × 80 mm Copernic balloon catheter (Balt Extrusion, Montmorency, France) to protect the hepatic artery supplying the transplant liver which arose just proximal to the aneurysm neck. Preservation of flow within the splenic artery and hepatic artery was maintained. No immediate complications were observed. Follow up CTA demonstrated persistence of the aneurysm with sub-optimal proximal stent wall apposition, not unexpected given initial difficulties with deployment. Repeat treatment with a second CASPER stent was subsequently performed at 12 months which resulted in complete aneurysm thrombosis on CTA performed 1 month later.

### Patient 3

**Fig. 3:**
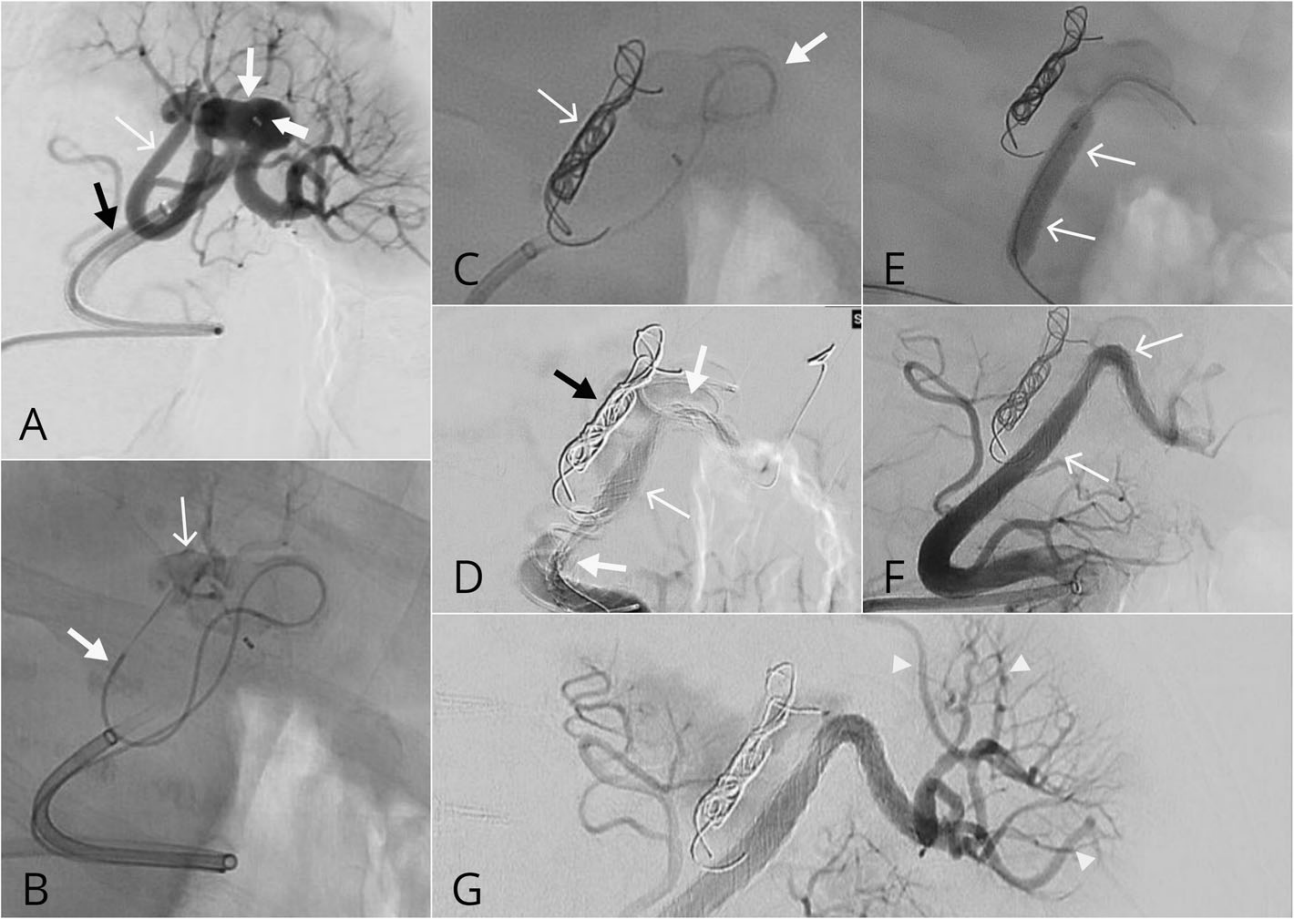
Complex distal splenic artery anerysm. **a** Initial angiography demonstrated a wide necked bilobed splenic artery aneurysm (thin white arrow) arising at a bifurcation and incorporating 2 outflow arteries. Note the presence of a 6Fr NeuronMAX 088 guide sheath (black arrow) and 5Fr Sofia (thick short white arrow) within the lateral lobe of the aneurysm. The medial outflow artery (thinnest white arrow) was targeted for sacrifice. **b** using a microcatheter (thick white arrow) the medial branch was coil sacrificed using detachable Target 360 XL coils. Note the aneurysm (thin white arrow). **c** following coil deployment (thin white arrows). **d** Overlapping CASPER stents (white arrows) were subsequently deployed across the aneurysm neck. Note two points of stent narrowing proximally and distally (thick white arrows) with ongoing occlusion of the medial branch (black arrow). **e** Angioplasty using an 8 × 40 mm Armada balloon (white arrows) resulted in **f** marked improvement of luminal diameter and angiographic appearances. **g** Distal splenic branches remained patent (arrowheads)

A 52-year-old female with an incidentally detected 2.8 cm bilobed splenic artery aneurysm.. Due to significant patient anxiety the procedure was performed under general anaesthetic. A 7-French vascular introducer sheath access was obtained via a rightfemoral approach. Initial splenic artery angiography demonstrated a large wide necked bilobed splenic artery aneurysm arising at a bifurcation and incorporating outflow arteries (Fig. 3a). The medial branch arising from the bilobed splenic aneurysm was coiled with a 10x40mm coil (Target XL 360 Stryker, MI, USA) to prevent persistent filling following treatment (Fig. 3b and c). The lateral branch supplying the largest portion of the spleen was preserved using two 8x40mm CASPER stents to exclude flow into the aneurysm. Significant tortuosity resulted in stent kinking which improved following balloon angioplasty using an 8 × 40 mm Armada balloon (Fig. 3d and e). The final angiogram demonstrated satisfactory position of the stent and preservation of flow distally (Fig. 3f and g). There were no procedural complications. Unfortunately, there was delayed septic splenic infarction 1 month following the procedure and the patient subsequently underwent a surgical splenectomy, This was favoured to relate to splenic artery tortuosity and probable thromboembolic complications related to points of stent narrowing despite initial improved angiographic appearances.

### Patient 4

A 58-year-old male with an incidentally detected 31 mm aneurysm of the distal right renal artery. Multiple segmental branches to the artery arose from the proximal aneurysm sac)*.* Under conscious sedation, right femoral artery access was obtained using an 8Fr sheath (Terumo, Tokyo, Japan). A 6-French Neuron-MAX 088 guide catheter was advanced into the right renal arteryand a 5Fr Sofia intermediate catheter was(MicroVention, CA, USA) advanced across the aneurysm neck into the outflow segmental artery. Following administration of 7000 IU of intra-arterial heparin, a 9 × 30 mm CASPER stent was deployed across the aneurysm neckfrom an interpolar artery with resultant partial aneurysmal flow stagnation (). 3.5 mg of Tirofiban was given intra-arterially to reduce the risk of early thromboembolic complications during stent deployment. No immediate complications were observed. Dual antiplatelet therapy with aspirin and clopidogrel was maintained at discharge. A CTA 2 months post-procedure showed a patent right renal artery with reduction in aneurysm size from 31 mm to 24 mm in diameter. There was partial thrombosis of the aneurysm sac with no thromboembolic complication. Follow up ultrasound demonstrated no flow within the aneurysm and patency of the parent arteries.

### Patient 5

A 55-year-old female presented with intermittent abdominal pain. CTA revealed a large 21x24mm saccular aneurysm at the left renal artery bifurcation with the anterior and posterior segmental branches arising from the proximal aneurysm neck. Under conscious sedation, right femoral access was obtained using an 8Fr sheath (Terumo, Tokyo, Japan). A 6-French Neuron-MAX 088 guide catheter was inserted into the left renal artery A 6Fr Sofia microcatheter (MicroVention, CA, USA) was then advanced across the aneurysm neck and a 7x18mm CASPER stent was deployed. There was some degree of stagnation of flow within the aneurysm on immediate post-treatment angiography. Resultant distal arterial vasospasm was treated with 5 mg IA Verapamil. There were no immediate complications. Subsequent ultrasound and 12 month CTA confirmed parent artery patency without evidence of in-stent stenosis. Unfortunately, the aneurysm remained patent on 12-month CTA with only a small volume of mural thrombus despite overall size reduction.

**Fig. 4:**
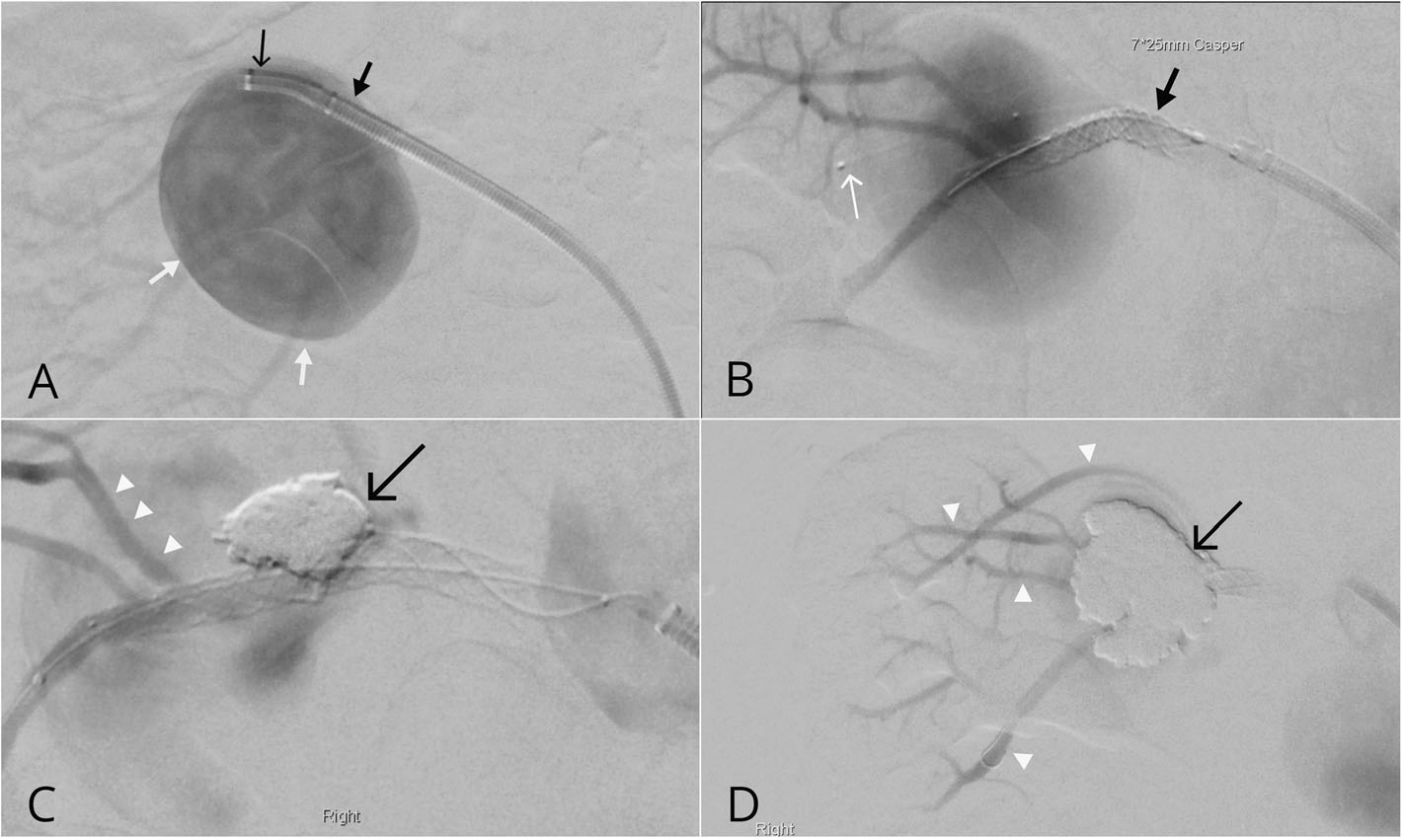
Combined CASPER stenting and Onyx embolisation. **a** A large right renal artery aneurysm (white arrows) was accessed with a guidesheath (thick black arrow) and inner diagnostic catheter (thin black arrow). **b** With the guidecatheter retracted proximally) a 7 × 25 mm CASPER stent (black arrow) was subsequently deployed over the aneurysm neck with a Rebar-14 microcatheter (white arrow) jailed within the aneurysm. Note mild contrast stasis. **c** A significant inflow jet was observed and as such a small volume of Onyx HD 500 (black arrow) was slowly injected to promote thrombosis. **c** and **d** Note all the segmental renal artery branches remained patent with near complete stasis and occlusion observed at the conclusion of the procedure

Patient 6A 77-year-old female had an incidentally detected large right renal artery aneurysm measuring 49 mm projecting inferiorly from the renal artery bifurcation which was increasing in size. Mid and lower pole segmental renal arteries arose from the aneurysm. The procedure was performed undergeneral anaesthetic due to perceived difficulties tolerating a prolonged procedure with conscious sedation. Right femoral access was obtained using a 9Fr sheath (Terumo, Tokyo, Japan). A quadraxial catheter system was used to access segmental branches of the right renal artery, through the neck of the large aneurysm (Fig. 4a and b). A 7 × 25 mm CASPER stent was deployed while a Rebar-14 was jailed within the aneurysm (Fig. 4b). 3 mg of IA Tirofiban was infused prior to injection of Onyx HD 500 (EV3 Inc., Plymouth, MI, US) into the aneurysm to decrease a significant residual inflow jet to facilitate aneurysm thrombosis. Subsequently there was a significant decrease in aneurysmal flow with stagnation on angiography (Fig 4c and d). CT angiogram 6 weeks post procedure demonstrated near complete thrombosis of the aneurysmal sac with a small residual patient component, patent renal artery and good parenchymal enhancement of the right kidney. A 3-month CTA revealed complete aneurysm thrombosis.

All procedures were initially technically successful despite parent vessel tortuosity with preserved flow to the distal parent artery following deployment. While there were no immediate complications a single case required 3 overlapping stents, one case required delayed re-intervention and unfortunately a single stent resulted in splenic infarction and splenectomy. All patients were maintained on dual antiplatelet therapy with low dose aspirin to continue indefinitely.

## Discussion

Incidence of VRAAs is increasing due to widespread use of cross-sectional imaging, as well as a rise in invasive procedures resulting in iatrogenic aneurysms. Prophylactic treatment for incidental aneurysms is favourable in situations where there is a high-risk of rupture given the potential for high morbidity and mortality. Excellent technical success rates with 90–95% of VRAAs able to be excluded with preservation of afferent arteries using an endovascular approach (Loffroy et al. 2015), also means that the risk-benefit ratio of treatment has shifted allowing treatment in more complex morphologies including those with wide necks or significant parent artery tortuosity.

Increasingly, endovascular therapy has been used as first-line treatment for VRAAs. Benefits include potential avoidance of general anaesthesia, shorter hospital stay, lower complication rates, improved visceral organ preservation and reported high technical success rates (Belli et al. 2012; Kok et al. 2016).

Possible complications of endovascular treatment include end-organ infarction (which may be clinically insignificant), arterial dissection, thrombosis, aneurysm or parent artery rupture, access complications such as groin haematoma or pseudoaneurysm, and major cardiac or cerebrovascular thromboembolic events. Published re-intervention rates due to delayed recurrence or aneurysm enlargement are low (4.4%) (Kok et al. 2016).

Conventional approaches with stent graft repair are complicated by the potential for kinking and injury of the parent artery with stiffer delivery systems, difficulty with accessibility of parent vessels and difficulties with adequate stent wall apposition and endoleak in tortuous anatomy (Sachdev-Ost 2010). In the past, repair utilising stents was hindered by tortuosity of the arteries, especially the splenic artery. However, reductions in available device diameters and improvements in flexibility make stenting an increasingly viable option.

Flow-diversion is a newer technique which can be utilised for VRAAs. Flow diversion is commonly utilized in neurointervention for intracranial aneurysms to reduce aneurysm inflow and outflow resulting in progressive stasis and thrombosis of the aneurysm sac. In VRAA, flow diverter stents are potentially useful for aneurysms with wide necks or that cannot be managed with covered stent placement because ofinsufficient landing zones within tortuous anatomy. This is especially important in distal renal artery aneurysms which occur in proximity to the bifurcation where flow-diversion is particularly valuable in preserving side-branch patency (Loffroy et al. 2015). Recent reports of flow diversion for VRAA and peripheral aneurysms demonstrate technical feasibility and early safety with aneurysm thrombosis reported in up to 90.6% to 98.5% of cases with aneurysm volume reduction in up to 82.7% of cases (Colombi et al. 2018; Maingard et al. 2019; Sfyroeras et al. 2012). Reported complication rates are low with 8.3% rate of acute stent thrombosis (Colombi et al. 2018; Maingard et al. 2019; Sfyroeras et al. 2012).

**Fig. 5:**
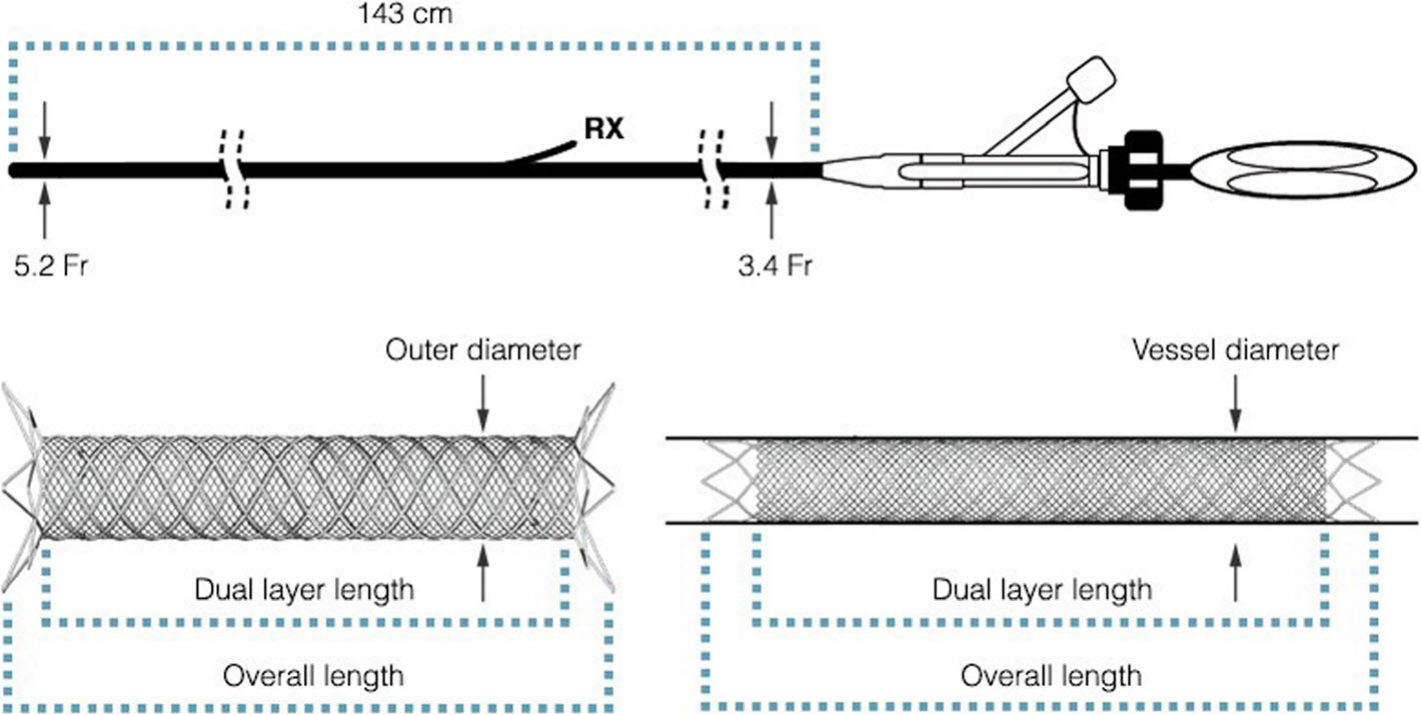
Unconstrained and deployed CASPER stents demonstrating the dual layer design and working length. The stent comes on a 5.2Fr 143 cm deployment shaft with a rapid exchange (RX) system allowing for improved navigability into tortuous anatomy

The CASPER stent provides a suitable alternative to flow diversion (Fig. 5). While not certified as a flow diverter the stent acts to reduce intra-aneurysmal flow in a similar mannerIt is designed as a braided, dual layer micromesh nitinol stent designed to prevent embolization of plaque in the treatment of carotid artery stenosis). The inner mesh has a smaller cell size of 375-500 μm to prevent embolic release from mural atheroma, while the braided nitinol design aims to minimize kinking and improve conformability to arterial anatomy. This is optimal for preventing embolization to distal organs in VRAAs but also provides flow diverting properties not dissimilar to certified flow diverters used to treat intracranial aneurysms where increased metal coverage reduces inflow jets and causes significant aneurysm sac thrombosis. A recent report by Akkan et al. reports successful treatment in 3 visceral aneurysms with the Roadsaver stent with successful obliteration on follow up imaging without reported complications (Akkan et al. 2017). Additional benefits for its use in VRAA include availability of larger stent sizes compared to conventional flow diverters which may be more suitable in larger splenic arteries with increased flexibility compared to covered stents, a potential advantage in tortuous or loop arteries, improved stent delivery and accuracy of deployment and improved wall apposition with the ability to use smaller 5Fr access depending on parent vessel tortuosityreducing post-procedure groin complications. In addition to this, the stent can be repositioned until up to 50% has been unsheathed. Importantly, this stent allows for side-branches to be preserved.

Its properties are offset by the reduced rates of early aneurysm occlusion compared to covered stents and increased risk of early thrombosis compared to uncovered stents used during stent assisted coiling due to increased metal coverage in its dual micromesh design.

Our preliminary experience with the CASPER stent for treatment of VRAAs shows that this is a technically feasible and relatively safe technique, with successful deployment and patency of the parent artery preserved during the procedure in all cases and immediate reduction of flow in all treated aneurysms. Aside from a single case, follow-up imaging demonstrated durable preservation of parent arterial patency and either reduction in size, partial or complete thrombosis of the aneurysms. Unfortunately, a single patient suffered a delayed 1 month large infected splenic infarct necessitating surgical splenectomy which was felt to most likely relate to thromboembolic complications due to multifocal stent narrowing which was initially managed with post deployment angioplasty with an initial good angiographic result.

The expectation when deploying a flow diverter is to see either slight flow reduction on the post deployment angiogram or no discernible difference in aneurysm flow. This was observed in all of the treated aneurysms in this series. Completion angiography is mostly useful to demonstrate patency and flow in the parent vessel and its branches, stent position and stent apposition to the vessel wall. A CTA at 6 weeks post procedure should show partial thrombosis and a reduction in aneurysm size and a 6-month post procedure CTA should show occlusion of the aneurysm in most cases although complete aneurysm occlusion can take upwards of 12 to 18 months depending on parent vessel morphology, aneurysm size and the utilisation of antiplatelet medications or anticoagulation. If there is persistent high flow and no reduction in aneurysm size at 6 to 12-month follow up, consideration should be given to deploying a second stent to further reduce inflow and promote stasis.

Deployment of the CASPER stent can be combined with advanced access techniques to access tortuous peripheral visceral arteries. The balloon anchoring technique was utilised in case 1 in order to advance the 6Fr NeuronMAX 088 into the mid to distal tortuous splenic artery. This is a commonly used technique in neurointervention. For example, the Flowgate balloon guide catheter (Stryker, MI, USA) can be used in order to anchor into distal tortuous vessels to provide a scaffold to either straighten the proximal catheter itself through proximal arterial tortuosity or through a difficult aortic arch or to advance more flexible distal access catheters without losing position. Similarly, within the intracranial vasculature, this can be performed with dual lumen balloon microcatheters to anchor distally while advancing distal access catheters to a more distal and stable position for intracranial aneurysm coil embolization or during endovascular thrombectomy during stroke. Significant tortuosity in case 1 was overcome using this technique using a 8x40mm Armada balloon to provide a scaffold to advance the 6Fr Neuronmax 088 guide sheath (Fig. 2). This technique can be used for difficult tortuous access in many peripheral interventions.

We routinely use dual antiplatelet therapy in patients in whom CASPER stents will be deployed to reduce the risk of acute stent thrombosis. Our practice is to utilise 7 days of 100 mg aspirin and 75 mg clopidogrel prior to the procedure. Intra-arterial glycoprotein IIb/IIIa inhibitors can be used as a rescue therapy for acute stent thrombosis intra-procedurally. Dual antiplatelet therapy is routinely continued following stent deployment for at least 3 months to reduce the risk of early in stent stenosis.

There were some limitations associated with using the CASPER stent. In case 2, the stent would not re-sheath for repositioning due to excess splenic artery tortuosity resulting in poor wall apposition and persistent flow at 12 months requiring a repeat procedure. Following a second CASPER deployment the aneurysm was completely occluded at 1 month. Use of the CASPER stent can be restricted due to limited size ranges, where the diameter is too small for some splenic arteries and the 40 mm length limit too short. Additionally, the cost associated with parent vessel reconstruction due to the cost of the CASPER stent itself and the need for additional guide sheaths and intermediate catheters should be weighed against parent artery sacrifice using conventional coiling which can be technically less challenging and significantly cheaper.

This series is limited by the variability in imaging follow up modalities with 2 patients followed up at external institutions with duplex Doppler ultrasound rather than CTA.

## Conclusion

The endovascular treatment of VRAAs is technically feasible using the dual-layer carotid CASPER stent with some promising results. Further larger series with longer follow-up are required to establish the durability of this technique.

## Data Availability

Data sharing not applicable to this article as no datasets were generated or analysed during the current study.
